# Comparative acute toxicity of intravenous paclitaxel and sirolimus in rats

**DOI:** 10.1016/j.crtox.2025.100248

**Published:** 2025-06-26

**Authors:** Jing Xie, Denise Schuett, Ulrich Speck, Tobias Haase

**Affiliations:** a Department of Radiology, Charité–Universitätsmedizin Berlin, corporate member of Freie Universität Berlin, Humboldt-Universität zu Berlin, and Berlin Institute of Health, Berlin, Germany; b InnoRa GmbH, Berlin, Germany

**Keywords:** Paclitaxel, Sirolimus, Intravenous, Toxicity, Rats

## Abstract

**Aim:**

In view of the exploration of sirolimus (rapamycin) as balloon coating for peripheral intravasal treatment and the reports on unfavorable tolerance of daily low-dose sirolimus, the aim of the study was to investigate potential toxicological effects of a single intravascular dose of sirolimus in comparison to paclitaxel and vehicle in the rat.

**Methods:**

Rats were treated intravenously with a single dose of 20 mg/kg sirolimus or paclitaxel dissolved in identical vehicle and sacrificed 5- or 14-days post treatment. Vehicle (Cremophor EL/Ethanol diluted with saline) treated rats served as a control. Potential effects on survival, hematology, organ weights, organ histology were analyzed.

**Results:**

Single dose i.v. injection of sirolimus, paclitaxel and vehicle induced temporary sedation after treatment. One animal treated with paclitaxel died, probably due to solvent toxicity. Sirolimus, paclitaxel and the vehicle control were tolerated. Animals treated with sirolimus or paclitaxel showed temporary hematological effects and thymic atrophy that subsided after 14 days. Sirolimus induced a temporary weight reduction of ovaries and uterus. Male rats showed histological changes of testes at 14 days after sirolimus treatment. Notably, sirolimus induced a prolonged body weight reduction compared to paclitaxel and vehicle treatment in male rats.

**Conclusion:**

Both substances showed similar and acceptable tolerability after high single-dose intravenous treatment. The results of this study do not indicate safety concerns that would preclude the use of sirolimus as an active ingredient on balloon catheters as an alternative to paclitaxel.

## Highlights

• Intravenous sirolimus and paclitaxel led to reduced thymus weights, accompanied by decreased white blood cell counts, consistent with their known mechanisms of action.
• Both drugs demonstrated similar and acceptable tolerability profiles, with no evidence to contraindicate sirolimus as an alternative to paclitaxel for high-dose endovascular applications.
• Sirolimus induced a prolonged reduction in body weight in male rats, a known metabolic effect that may offer potential benefits for patients with cardiovascular diseases.

## Introduction

Paclitaxel (PTX) and sirolimus (SRL) are both approved drugs for use in cancer therapy or immunosuppression, respectively. In addition to these applications, both drugs serve as proliferation inhibitors on drug-eluting stents (DES) for the treatment of stenotic coronary vessels, typically delivered at doses of 60–150 µg per stent. Higher doses, up to 10 mg of PTX, have been employed on drug-coated balloon (DCB) catheters for peripheral vascular applications. Despite decades of successful use, retrospective analyses of clinical data revealed an association between paclitaxel-coated medical devices for peripheral use and increased rates of mortality and limb amputation (Katsanos, 2018, Katsanos, 2020). This prompted the U.S. Food and Drug Administration (FDA) to issue a warning in January 2019 regarding paclitaxel-coated devices, which subsequently spurred research into limus-coated balloon alternatives. Although the FDA has recently revised its warning on PTX, a positive attitude for SRL in endovascular treatments persisted within the scientific community. This is partly supported by early cell culture studies reported by Wessely *et al.* (Wessely et al., 2006), which seem to indicate a higher toxicity and narrower therapeutic window for PTX compared to SRL. On the other hand, clinical trials have highlighted a poor tolerability of repeated oral doses of SRL (Burkhalter, 2012, Waksman, 2004, Smith, 2008). Given these findings, it is important to assess whether the high intravascular doses of SRL required for peripheral arteries via DCB are tolerated. While intravenous toxicities of PTX are documented by Kadota *et al*. (Kadota, 1994, Kadota, 1994), data for limus-drugs ranges, to the best of our knowledge, from a calculated LD50 of single dose 6.3  mg/kg (rat i.v., (Assessment Report Votubia, 2011) to overt lethality at 10 mg/kg (rat i.v., (Yanez, 2008) to rats that survived up to 250  mg/kg (rat i.v., (Rapamune, 1999). To support studies on limus-drugs as coatings for endovascular devices in high-dose peripheral applications, it is essential to establish comparable toxicological profiles for parenteral PTX and SRL. Based on uncertain SRL tolerability and known PTX tolerability up to 65 mg/kg (rat single dose i.v. (Kadota, 1994), a pilot dose-finding study with 0.2, 2, and 20 mg/kg was done that confirmed no drug related deaths at these doses. Afterwards, we straightforwardly compared the tolerability of 20 mg/kg SRL versus PTX and the corresponding drug-vehicle in an intravenous single-dose toxicity study in rats.

## Materials and methods

### Chemicals

For the preparation of the test solutions the following chemicals were used: butylhydroxytoluol (BHT) (Applichem, A3777,0100, Lot# 8G001385), Cremophor EL (CrEL) (Sigma, product named Kolliphor EL, C5135-500G, Lot# BCBZ9384), ethanol (EtOH) (J.T. Baker Ethanol Absolute, 8025, Lot# 1828901875), sirolimus (Fujian Kerui Pharmaceutical Co Ltd., Lot# 140201), paclitaxel (Hande Bio-Tech, Lot# ZCP-130201).

### Preparation of test solutions

240 mg of SRL and PTX were weighed and dissolved in 10 ml ethanol each with 2.4 mg BHT (1 % m/m active substance). BHT was added as antioxidant to reduce SRL oxidation. To yield comparative solutions of both compounds, BHT was also added to paclitaxel and control solution. The solutions were vortexed for dissolution for approx. 2 min. Then 10  ml Cremophor EL were added and vortexed to obtain 20  ml solution (nominally 12  mg/ml concentration). Twenty ml of vehicle were prepared by mixing 10 ml ethanol with 2.4 mg BHT (accordingly to 1 % m/m in samples with active substances) with 10 ml Cremophor EL. The solution was vortexed for 2 min. All solutions were clear with a slightly yellowish appearance.

The pH of the stock solutions (12 mg/ml and vehicle) was analysed using colorimetric pH test strips. HPLC analyses confirmed a PTX concentration of 12.12  mg/ml (method see (Gemeinhardt, 2021) and a SRL concentration of 10.80 mg/ml (Analytik Kirchhoff GmbH, Berlin). All stock solutions (including vehicle) were diluted 1:10 with 0.9 % NaCl Injection (B. Braun, Melsungen) immediately before administration.

### Animal study

All animal studies were conducted at LPT Laboratory of Pharmacology and Toxicology GmbH & Co. KG, 21,147 Hamburg, Germany in compliance with the “Good Laboratory Practice” Regulations and carried out in accordance with the Standard Operating Procedures (SOPs). Male and female Wistar Han rats were obtained from Charles River Laboratories Germany GmbH. Rats (aged 59 days) were intravenously infused (dose / over 6 min) via the tail vein with SRL/PTX solutions or vehicle. On the day of the experiment, the male rats weighed 220–255 g and the female rats weighed 151–184 g.

Since there were no toxicological data for SRL in Cremophor EL/ethanol and only limited data for PTX (Kadota *et al.* 1994 I and II) available, a pilot study with increasing SRL or PTX at 0.2 mg/kg, 2 mg/kg, or 20 mg/kg and an observation period of 8 days was carried out in 12 rats (2 each per drug and dose).

**Fig. 1:**
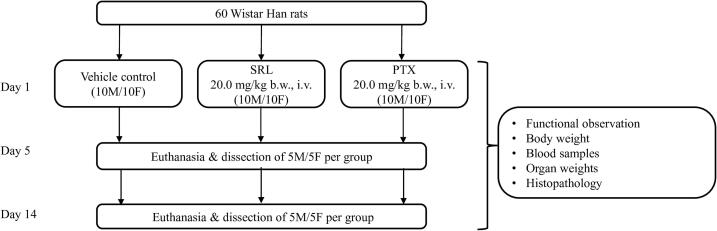
Schematic flowchart of study design. M = male; F = female. Vehicle = Cremophor EL/ethanol (1:1) diluted 1:10 in 0.9 % saline solution.

For the single dose toxicity study, rats were randomized into 3 groups of 20 animals (10 male, 10 female rats per group) and given the treatment as shown in Fig. 1. The first day of administration was recorded as day 1.

### Evaluation

The animals were observed individually before and after dosing for behavioral changes and reactions to treatment. Special attention was paid to the local tolerance at the injection sites. The body weight was measured and recorded before administration and on the day of sacrifice and dissection. Blood samples were collected from the retrobulbar venous plexus under isoflurane anaesthesia from animals fasted overnight for blood counts and haematological analysis. On day 5 and day 14, animals were euthanised by CO_2_ inhalation, exsanguinated, weighed, dissected and inspected macroscopically. All tissues were examined visually. The epididymis, kidney, liver, ovary, prostate, seminal vesicle, spleen, testicle, thymus and uterus were weighed and preserved for pathological examination. The section “Histopathological findings” summarizes observations considered non-incidental. All other microscopic findings are considered incidental, unrelated to the test compounds, spontaneous in nature, and consistent with the normal background pathology observed in rats of this strain and age.

### Statistical analysis

Toxicology and pathology data were captured, whenever possible, using the LPT departmental computerised systems (Provantis® Integrated preclinical software, Instem LSS Ltd., version 10.2.1). Homogeneity of variances and normality of distribution were tested using the BARTLETT’s and SHAPIRO-WILK’s test. If heterogeneity was observed in a set of data, stepwise transformation of the values into logarithmic or rank values was performed prior to ANOVA. If the ANOVA yielded a significant effect (*p* ≤ 0.05), intergroup comparisons between groups were made by the DUNNETT’s test (*p* ≤ 0.01 and *p* ≤ 0.05). Statistical analysis was performed using the GraphPad Prism 8.4 software (San Diego, USA).

## Results

### Drug tolerability

In the exploratory pilot study, injection of PTX and SRL showed no local intolerance, no test-item related systemic intolerance and no macroscopic post-mortem changes. Rats in the highest dosage groups (20 mg/kg b.w.) showed temporary (lasting for max. 1 h) abdominal position, increased respiratory rate and reduced motility. Following an initial reduction in weight gain in the 20  mg/kg groups, all rats gained weight over the 8-day observation period (see supplementary Fig. S1).

**Fig. 2:**
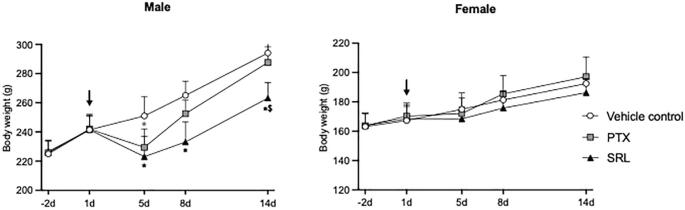
Mean and standard deviation of body weight over time in acute toxicology experiment of male and female rats treated with vehicle control, PTX and SRL as a single-dose infusion (arrow). Significant differences versus vehicle control (*p ≤ 0.01) and PTX (^$^p ≤ 0.05) at the same time point are shown.

In the following single-dose toxicity study, intravenous injection of 20  mg/kg PTX, SRL or the corresponding volume of the vehicle caused no local intolerance at the injection site. Rats in all groups showed temporary sedation after dosing for max. 6 h. One rat in the PTX group died within 3 h after injection. The body weight of male rats treated with PTX was temporarily decreased by up to 9 % compared to the vehicle control (Fig. 2). This difference subsided within the observation period of 14 days. The body weight of male rats treated with SRL was decreased by 11 % to 12 % until the end of the observation period (Fig. 2). Overall (including females), there was no statistic reduction in body weight on day 14 compared to day 1 observed across all groups.

### Hematological analysis

**Fig. 3:**
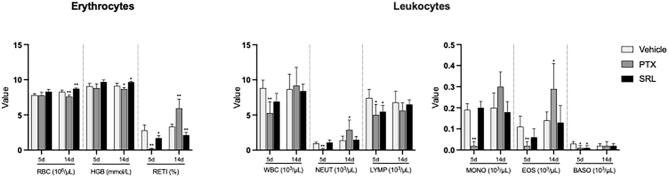
Hematological parameters of male rats treated with vehicle control, paclitaxel (PTX) and sirolimus (SRL) as a single dose infusion. Values are expressed as mean ± SD. Significant differences versus vehicle control at the same time point are shown as *p ≤ 0.05; ** p ≤ 0.01. RBC − red blood cells; HGB − hemoglobin; RETI − reticulocytes; WBC − white blood cells; NEUT − neutrophils; LYMP − lymphocytes; MONO − monocytes; EOS − eosinophils; BASO − basophils.

A temporary decrease of differential blood count and reticulocytes was observed in both treatment groups for male rats compared to the vehicle control group on day 5 (Fig. 3 and supplementary Table S2). This reduction was more pronounced in the PTX group compared to the SRL group and subsided for most of the parameters on day 14.

**Fig. 4:**
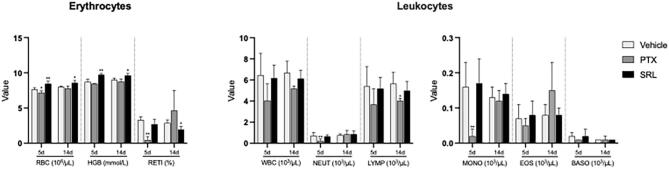
Hematological values of female rats relative to start date treated with vehicle control, PTX and SRL as a single dose infusion. Values are expressed as mean ± SD, *p ≤ 0.05; **p ≤ 0.01. RBC − red blood cells; HGB − hemoglobin; RETI − reticulocytes; WBC − white blood cells; NEUT − neutrophils; LYMP − lymphocytes; MONO − monocytes; EOS − eosinophils; BASO − basophils.

In female rats PTX injection led to a temporary reduction in reticulocytes, neutrophils, and monocytes on day 5 that subsided on day 14 (Fig. 4 and supplementary Table S3).

### Macroscopic findings

On day 5, a reduced size of the thymus in all male and female animals treated with PTX and in 2 of 5 male and 3 of 5 female animals treated with SRL was evident. At macroscopic examination on day 14, only in 1 of 5 male animals treated PTX a smaller thymus was seen. No macroscopic findings were noted for the female animals on test day 14.

### Organ weights

**Fig. 5:**
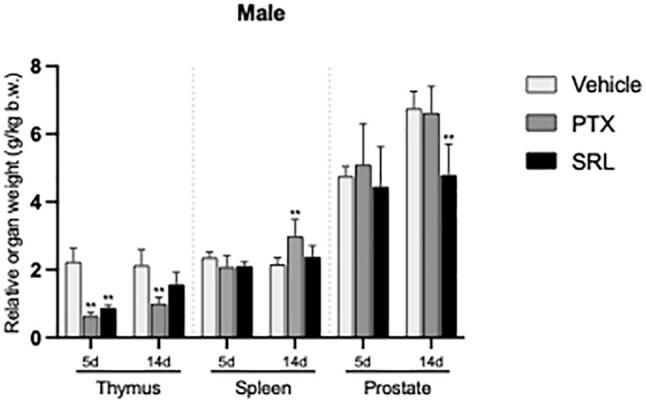
Relative organ weights (g/kg b.w.) of male rats. Values are expressed as mean ± SD. Significant differences versus vehicle control at the same time point are shown as *p ≤ 0.05; ** p ≤ 0.01.

**Fig. 6:**
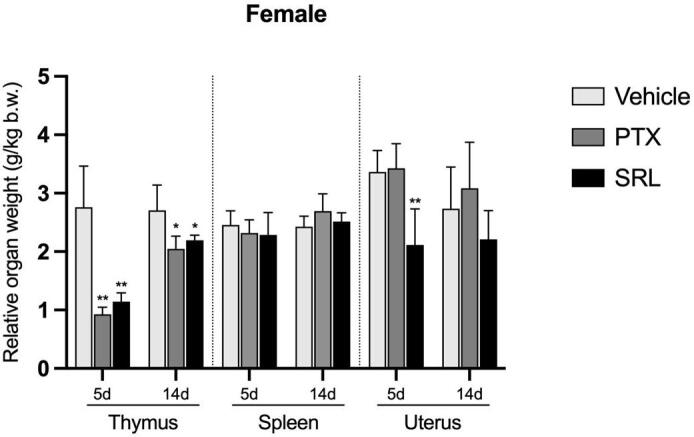
Relative organ weights (g/kg b.w.) of female rats as a single dose infusion. Values are expressed as mean ± SD. Significant differences versus vehicle control at the same time point are shown as *p ≤ 0.05; ** p ≤ 0.01.

On day 5, rats treated with PTX and SRL showed reduced relative weights of the epididymis, pituitary gland, spleen and thyroids, although these reductions did not reach statistical significance (see supplementary Table S4, 5). Notably, the relative thymus weight was significantly decreased (*p* ≤ 0.01) compared to the vehicle control (Fig. 5, Fig. 6). Additionally, the relative weights of the ovaries (no significant difference) and uterus (statistically significant at *p* ≤ 0.01) were decreased in the animals treated with SRL (Fig. 5, Fig. 6 and supplementary Table S4, 5). Except for the weight of thymuses in PTX and SRL and prostate/seminal vesicles in SRL treated animals, all organ weights showed a normalization on day 14 (see supplementary Table S4, 5).

### Histopathological findings

Nearly all PTX- and SRL-treated rats but none of the animals treated with the vehicle showed atrophic changes in the bone marrow (os femoris) on day 5 that subsided on test day 14. Atrophy of the testes of 3 of 5 animals, oligospermia in the epididymis of 1 of 5 animals and spermatid giant cells in the testes of 3 of 5 animals were noted in the males treated with SRL on day 14. Histopathological changes in the kidneys compared to naïve tissues were evident in form of minimal to mild lymphocytic infiltration, tubular basophilia, tubular dilation, tubule hyaline cast and tubule epithelial degeneration/necrosis in some male and female animals in vehicle control-, and drug-treated groups. These changes subsided on day 14.

## Discussion

Intravenous treatment of rats with 20 mg/kg b.w. PTX and SRL was tolerated with temporary effects on differential blood count, reticulocytes, and thymic atrophy. These observations are in accordance with the pharmacologic actions of both drugs (Fetterly et al., 2001, Hong and Kahan, 2000, Wang, 2016, Wang, 2016). Reduced motility after infusion and temporary histopathological changes in kidneys were observed in all groups (DiJoseph et al., 1992, Abraham, 1991) and are therefore due to the organic solvents in the vehicle. The effects of SRL on reproductive organs are likely due to blockage of mTOR regulated pathways (Guo and Yu, 2019, Schell, 2016), but were elsewhere shown to be reversible (Rovira, 2012). The prolonged weight loss observed in SRL treated male rats is not due to malaise, but rather a known effect on food intake and energy homeostasis (Hebert, 2014). Preclinical studies show that SRL induced mTOR inhibition affects metabolic control, significantly reducing weight gain in various species (Hebert, 2014, Chang, 2009, Ross, 2015). High SRL blood levels preferably achieved by parenteral routes are suggested to inhibit mTOR in muscles, liver, and the central nervous systems, leading to long-lasting metabolic effects (Hebert, 2014, Yang, 2012). A more pronounced weight reduction in male compared to female rats has also been observed in previous toxicological studies on SRL (Rapalimus, 2014). This effect may be attributed to the greater muscle mass in males, offering a larger metabolic target for mTOR inhibition (Gartych, 2021). Additionally, testosterone is known to upregulate mTOR activity in muscle tissue (White, 2013), which could enhance susceptibility in males, although this remains speculative.

Previous investigations in patients confirmed the anti-restenotic efficacy of SRL after oral administration (Waksman, 2004) however, shed doubt about its tolerance (Burkhalter, 2012, Smith, 2008, Kufner, 2009). Thus, considering the step from low dose coronary application to increased dose in peripheral arteries it seemed appropriate to investigate the tolerance after single dose intravascular use in experimental animals. Current information on the intravenous use of PTX and SRL are derived from cancer treatments. Both drugs display a similarly low water solubility which causes problems for testing in cell culture and intravascular administration. In tumor therapy PTX is applied in a dose of 175 mg/m^2^ body surface in a special preparation (Taxol™, Bristol-Myers Sqibb, Cremophor EL/ethanol 1:1 (v/v)) as intravenous infusion repeatedly with 3 weeks break between treatment cycles or as Abraxane (Abraxis Biosciences) for tumour therapy. SRL is commercially available for oral administration as an immunosuppressant given in a daily dosage of 2–10 mg (bioavailability 15 %, e.g., oral Rapamune, Pfizer) and as albumin-bound intravenous formulation (Fyarro) in a dose of 100  mg/m^2^ for cancer therapy. Toxicological studies on PTX formulated in Cremophor EL/ethanol have been published by Kadota *et al.* (Kadota, 1994, Kadota, 1994), whereas only a limited number of studies have investigated the effects of intravenously administered SRL in animal models, yielding variable results. Honcharik *et al.* analysed the pharmacokinetics of rapamycin after single-dose i.v. treatment in rabbits of 0.5 mg/kg showing no obvious toxicological effects (Honcharik et al., 1992). In a study on allograft rejection in rabbits, daily i.v. treatments with SRL (1 mg/kg, 60d), showed no effect on hepatic or renal function (Fryer, 1993). In a study by Yanez *et al*., 60 % of the rats died within 24 hrs. after 10 mg/kg single-dose SRL i.v. treatment. However, this study did not include a vehicle group (Tween80/PEG 400/DMA) (Yanez, 2008). Intravenous treatment with 10 mg/kg everolimus in rats was 100 % lethal (Assessment Report Votubia, 2011). In contrary, older studies on the toxicity of rapamycin report on the application of up to 250 mg/kg i.v. in rats that were not 100 % lethal (Rapamune, 1999). One potential explanation for the variable toxicities observed with intravenous SRL is the toxicity of the solvents used to dissolve the poorly water-soluble drug. Similar findings have been reported for the formulation of PTX in Taxol® (6 mg/mL PTX in Cremophor EL/ethanol), where the vehicle itself has been implicated as a significant contributor to the observed toxic effects (Kadota, 1994). While Cremophor EL/ethanol is not the clinically established solvent for SRL and may contribute to toxicological effects, identical vehicles were used for both compounds to enable clear differentiation between drug-specific and vehicle-related effects.

Considering peak PTX plasma levels after treatment with paclitaxel-coated balloons in peripheral arteries (Freyhardt, 2011) and the drug doses of SRL-coated balloons tested in clinical trials, future applications of SRL-coated balloons in peripheral arteries could reach a dose up to 0.35  mg/kg and clearly surpass tolerated doses after repeated oral administration. Although serum drug concentrations were not measured in our study, limiting pharmacokinetic evaluation, the initially infused dose of 20  mg/kg corresponds to a known human equivalent dose (HED) of 3.2  mg/kg (Nair and Jacob, 2016). This exceeds expected clinical exposure by approximately 10-fold providing a safety margin consistent with regulatory guidelines for toxicological risk assessment (Nair and Jacob, 2016, Lowe, 2010). Based on our results, intravascular delivery of SRL via coated balloons appears to offer a safety profile comparable to that of established paclitaxel-coated devices. A SRL induced reduction in body weight was recently observed in humans, suggesting the need for body weight monitoring in clinical studies involving high-dose SRL-coated balloon catheters (Mannaa, 2023).

## CRediT authorship contribution statement

**Jing Xie:** Data curation, Formal analysis, Writing – review & editing. **Denise Schuett:** Formal analysis. **Ulrich Speck:** Funding acquisition, Project administration, Writing – review & editing. **Tobias Haase:** Data curation, Supervision, Writing – original draft.

## Declaration of competing interest

The authors declare that they have no known competing financial interests or personal relationships that could have appeared to influence the work reported in this paper.

## Data Availability

Data will be made available on request.
