# Supplementary material for: Comparative acute toxicity of intravenous paclitaxel and sirolimus in rats
Source: Curr Res Toxicol. 2025 Jun 26;9:100248. doi: 10.1016/j.crtox.2025.100248 (PMC12271596; doi:10.1016/j.crtox.2025.100248)
Supplement: Supplementary Data 1 [file mmc1.docx]

**Xie et al. Supplement:**

Supplementary Figure S1: Exploratory pilot study: Mean body weight in male rats (n=2) treated with a single-dose injection (arrow) of PTX and SRL at 0.2 mg/kg, 2 mg/kg and 20 mg/kg, respectively. Following a transient decrease in mean body weight in the 20 mg/kg dosage groups, all rats gained weight over the observation period of 8 days.

Supplementary Table S2: Hematological parameters of male rats treated with vehicle control, paclitaxel (PTX) and sirolimus (SRL) as a single dose infusion. Values are expressed as mean ± SD. Significant differences versus vehicle control at the same time point are shown as *p ≤ 0.05; ** p ≤ 0.01.

Supplementary Table S3: Hematological parameters of female rats treated with vehicle control, paclitaxel (PTX) and sirolimus (SRL) as a single dose infusion. Values are expressed as mean ± SD. Significant differences versus vehicle control at the same time point are shown as *p ≤ 0.05; ** p ≤ 0.01.

Supplementary Table S4. Relative organ weights (g/kg b.w.) of male rats. Values are expressed as mean ± SD. Significant differences versus vehicle control at the same time point are shown as *p ≤ 0.05; ** p ≤ 0.01.

Supplementary Table S5. Relative organ weights (g/kg b.w.) of female rats. Values are expressed as mean ± SD. Significant differences versus vehicle control at the same time point are shown as *p ≤ 0.05; ** p ≤ 0.01.
